# Integrated Au-Nanoroded Biosensing and Regulating Platform for Photothermal Therapy of Bradyarrhythmia

**DOI:** 10.34133/2022/9854342

**Published:** 2022-02-07

**Authors:** Jiaru Fang, Dong Liu, Dongxin Xu, Qianni Wu, Hongbo Li, Ying Li, Ning Hu

**Affiliations:** ^1^State Key Laboratory of Optoelectronic Materials and Technologies, Guangdong Province Key Laboratory of Display Material and Technology, School of Electronics and Information Technology, State Key Laboratory of Ophthalmology, Zhongshan Ophthalmic Center, Guangdong Provincial Key Laboratory of Ophthalmology and Visual Science, Sun Yat-sen University, Guangzhou 510006, China; ^2^Stoddart Institute of Molecular Science, Department of Chemistry, ZJU-Hangzhou Global Scientific and Technological Innovation Center, Zhejiang University, Hangzhou 310058, China; ^3^Molecular Cancer Research Center, School of Medicine, Sun Yat-sen University, Shenzhen 518107, China; ^4^State Key Laboratory of Transducer Technology, Chinese Academy of Sciences, Shanghai 200050, China

## Abstract

Bradyarrhythmia is a kind of cardiovascular disease caused by dysregulation of cardiomyocytes, which seriously threatens human life. Currently, treatment strategies of bradyarrhythmia mainly include drug therapy, surgery, or implantable cardioverter defibrillators, but these strategies are limited by drug side effect, surgical trauma, and instability of implanted devices. Here, we developed an integrated Au-nanoroded biosensing and regulating platform to investigate the photothermal therapy of cardiac bradyarrhythmia *in vitro*. Au-nanoroded electrode array can simultaneously accumulate energy from the photothermal regulation and monitor the electrophsiological state to restore normal rhythm of cardiomyocytes in real time. To treat the cardiomyocytes cultured on Au-nanoroded device by near-infrared (NIR) laser irradiation, cardiomyocytes return to normal for long term after irradiation of suitable NIR energy and maintenance. Compared with the conventional strategies, the photothermal strategy is more effective and convenient to regulate the cardiomyocytes. Furthermore, mRNA sequencing shows that the differential expression genes in cardiomyocytes are significantly increased after photothermal strategy, which are involved in the regulation of the heart rate, cardiac conduction, and ion transport. This work establishes a promising integrated biosensing and regulating platform for photothermal therapy of bradyarrhythmia in vitro and provides reliable evidence of photothermal regulation on cardiomyocytes for cardiological clinical studies.

## 1. Introduction

The heart plays a vital role in the circulatory system to maintain the life activities by transporting blood throughout the body. The electrical conduction system of the heart can generate rhythmic action potentials by the autonomic pacemaker cells, driving the working cardiomyocytes to rhythmically contract and pump blood to other organs or tissues. However, some hereditary or acquired reasons, such as organic heart disease, drug abuse, severe electrolyte balance disorders, and cardiac electrical conduction disorders will induce cardiac arrhythmias as a huge group in cardiovascular diseases [1, 2]. Globally, cases of sudden cardiac arrest due to arrhythmia account for half of cardiovascular disease patients and 15% of death [3]. In China, 330 million cardiovascular diseases suffer from the arrhythmias, leading the main cause of cardiovascular death [4]. Arrhythmias can be categorized into tachyarrhythmias and bradyarrhythmias according to heartbeat rhythm. The bradyarrhythmias could be induced by sinoatrial node dysfunction, atrioventricular block, drug influence, etc., which often results in angina pectoris, cardiac insufficiency, or central nervous system dysfunction. Therefore, much effort is attempted to develop the effective bradyarrhythmia therapies, aiming at the rhythmical regulation of the heart [5].

At present, drug therapy is one of the main strategies to treat bradyarrhythmias and isoproterenol is the typical drug to increase the heart rate [6]. Moreover, surgery therapy is also an effective and successful strategy to treat the disease, but the large trauma and the high probability of postoperative bleeding limited are main limitations [7*–*9]. Meanwhile, implantable cardioverter defibrillators [10, 11] are accepted and implemented to pace the heart. Although these devices are effective to treat bradyarrhythmias, they are potential to contaminate the physiological environment of the thoracic cavity due to the immunogenicity or unpredictable electrochemical reactions at the device-tissue interface [12]. Recently, a large number of studies reported the optical regulation methods to recover the normal rhythm of cardiomyocytes, which serve as an alternative strategy to improve the implantable cardioverter defibrillators [13, 14]. Optogenetics is one of the promising approaches to activate ion channels by regulating the excitability of cardiomyoctyes [15]. Bruegmann et al. applied photosensitive protein channelrhodopsin-2 to regulate the cardiomyocyte beating [16]. Although optogenetics is effective and noninvasive, it is difficult to achieve cardiac regulation *in vivo* and clinically due to the gene transfection/modification. Some group proposed the nonoptogenetic regulation to regulate the cardiomyocytes by photostimulation with the nanomaterials. The optogenetic regulation can also trigger the action potential of cardiomyocytes [17, 18]. Smith et al. regulated the cardiomyocytes with a near-infrared (780 nm) femtosecond laser and monitored the intracellular calcium ion activities [19]. Dittami et al. applied a 1875 nm pulsed infrared diode laser to regulate a quail embryonic heart, leading to induced calcium oscillation and contraction of neonatal cardiomyocytes by heating effects [20]. Gentemann et al. proposed the concept of a light-based and nanoparticle-mediated stimulation system, demonstrating that irradiation of a 532 nm picosecond laser causes the calcium oscillations of cardiomyocytes to restore cellular activity [21].

Electrical excitability is an intrinsic physiological property of cardiomyocytes, and the changes in electrophysiological signals can be applied to evaluate the therapeutic effects on bradyarrhythmias [22, 23]. Currently, label-free and noninvasive extracellular recording techniques are widely applied to monitor the electrical activities of excitable cells over a long period of time [24–28]. Moreover, with the development of micro-/nanofabrication technologies, new micro-/nanoelectrode devices are developed to form a tight cell-electrode coupling [29–33]. The phototransduction coupling mechanism can be induced when cells are photostimulated on nanopatterned devices [34]. Although the phototransduction coupling mechanism is not fully understood, the evidence is provided to support the photothermal effect as the underlying mechanism [35–38]. The near-infrared (NIR) light from 700 nm to 950 nm is the suitable wavelength range to produce the photothermal effects. Noble metal nanoparticles (such as Au, Ag, and Pt) can absorb light energy at specific wavelengths and produce localized surface plasmon resonance (LSPR), and the LSPR band and heat production capability can be optimized by adjusting the size and morphology of metal nanomaterials [39, 40]. On the other hand, gold nanoparticles have the advantages of chemical inertness and good biocompatibility, which is a popular choice in the current photothermal therapy [41]. The absorption of photonics by the nanomaterial can cause the photothermal effect, which increases the temperature around the surface of nanomaterials and generates a thermal field, and this photothermal effect is successfully applied to pace and regulate the electrophysiological activities of cardiomyocytes [12, 42, 43]. For example, when the Au-3D nanomaterial device is irradiated by near-infrared laser irradiation, which matches the main absorption spectrum, it converts the optical energy into heat due to the plasma thermal effect. After the device has accumulated enough heat and the temperature increases to a high level, the proteins in the cardiomyocytes can be stimulated to respond [44]. Though photothermal effect is well-developed to pace the cardiomyocytes, the photothermal therpy is seldom reported to treat bradyarrhythmia.

**Figure 1:**
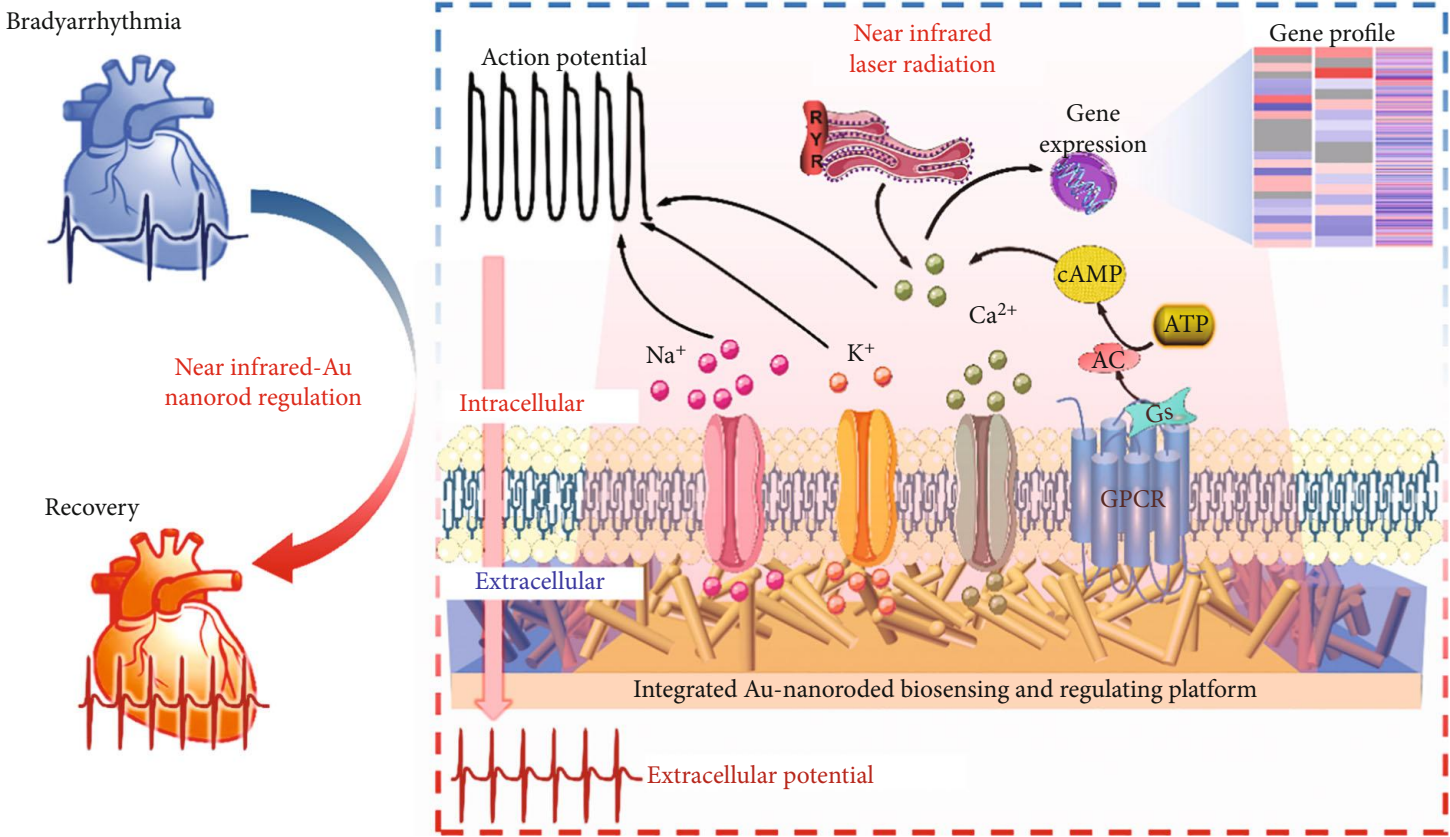
Integrated Au-nanoroded biosensing and regulating platform for the photothermal therapy of bradyarrhythmia. The cardiomyocyte cultured on the Au-nanoroded electrode array are regulated by near-infrared laser irradiation. The effect of photothermal regulation is assessed by extracellular potential recording and mRNA sequencing.

Herein, we developed an integrated Au-nanoroded biosensing and regulating platform to investigate the photothermal therapy of cardiac bradyarrhythmia in vitro (Figure 1). The Au-nanoroded electrode array can gently accumulate enough thermal energy and maintain a long period of repair time after completing the irradiation. After treating with near-infrared (NIR) laser irradiation, cardiomyocytes cultured on Au-nanoroded electrode array return to normal and maintain for a long period of time. Compared with the conventional strategy, this photothermal therapeutic strategy is more effective and convenient. Furthermore, mRNA sequencing shows that the differential expression genes (DEGs) in cardiomyocytes are significantly increased after photothermal regulation, focusing on ion channel genes, transporter genes, and Ca^2+^ regulatory protein genes. The DEGs are related with the heart rate, cardiac conduction, and ion transport. This work establishes a promising integrated biosensing and regulating platform for photothermal therapy of bradyarrhythmia *in vitro* and provides the reliable evidence of photothermal regulation on cardiomyocytes for cardiological clinical studies.

## 2. Results and Discussion

### 2.1. Establishment and Characterization of Integrated Au-Nanoroded Biosensing and Regulating System

**Figure 2:**
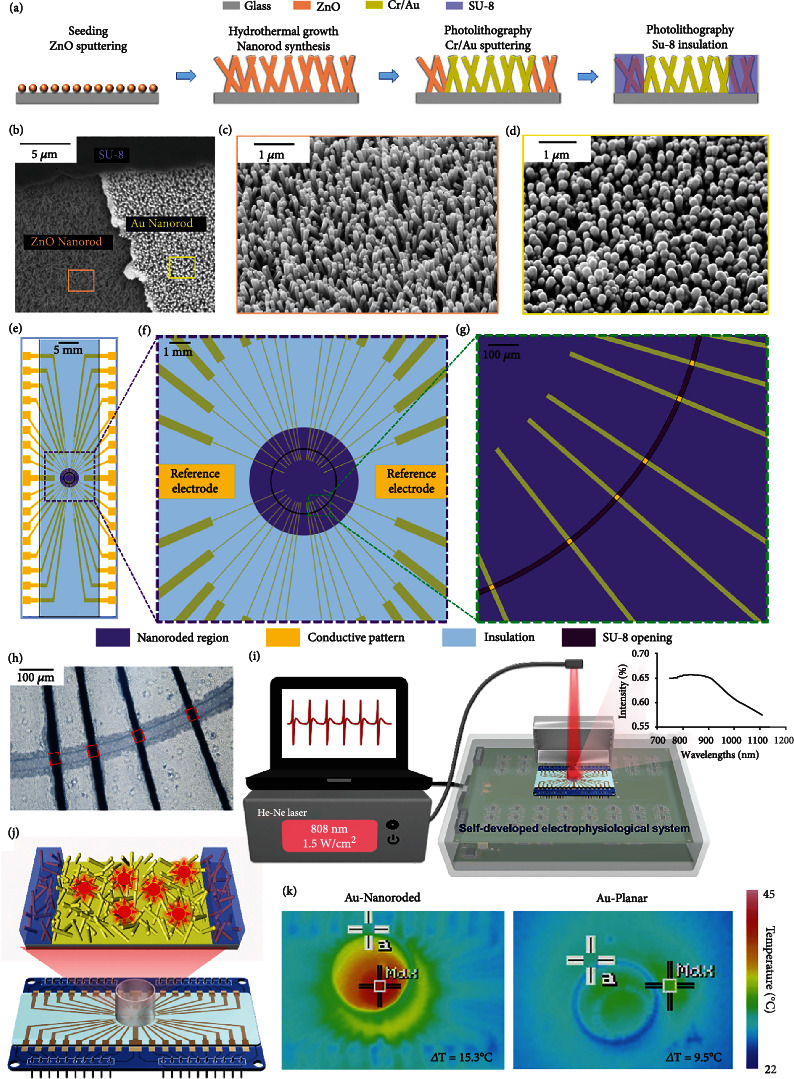
Integrated Au-nanoroded biosensing and regulating system. (a) Fabrication of Au-nanoroded device, including ZnO seeding, hydrothermal growth of ZnO nanorod synthesis, conductive layer pattern, and SU-8 layer insulation. (b–d) Scanning electron microscope (SEM) images of a nanoroded device with the ZnO-nanoroded region, Au-coated-nanoroded region, and SU-8 insulating region. Typical SEM images of ZnO nanorods (c) and Au-coated nanorods (d). (e–g) Layout of the device. (e) Overview of the layout showing the electrically 32 conductive recording electrode tracks and 2 conductive reference electrodes (yellow). (f) The enlarged view showing the layout of the nanoroded region (purple) with a diameter of 4 mm and recording sizes at the center of device. (g) 20 × 20 *μ*m^2^ recording electrodes at the SU-8 insulating opening. (h) Optical images showing Au-nanoroded MEA with a 20 *μ*m opening ring. The Au-nanoroded sites are indicated in red box. (i) Schematic of integrated Au-nanoroded biosensing and regulating system including 808 nm He-Ne laser, Au-nanoroded MEA device, and self-developed electrophysiological recording system. (j) Schematic of multichannel Au-nanoroded device and Au-nanorod-induced photothermal effect. (k) Thermal images of the Au-nanoroded electrode array (MEA) and Au-planar MEA showing the temperature change after radiation with a power of 1.5 W/cm^2^ for 5 min. The maximum temperature of Au-nanoroded MEA is 41.3°C, which is 15.3°C higher than that of edge area. The maximum temperature of Au-planar device is 31.9°C, which is 9.5°C higher than that of edge area.

The Au-nanoroded device is the key component of the integrated biosensing and regulating integrated system, and the device is fabricated by hydrothermal growth and standard photolithography (Figure 2(a)). The synthesis of ZnO nanorods consists of two steps: (i) ZnO seeding on the glass substrate by sputtering, and (ii) the synthesis of ZnO nanorods by hydrothermal growth. After ZnO nanorod synthesis, the Cr/Au (10 nm/100 nm) conductive layer is defined by photolithography and patterned on the ZnO-nanoroded region by sputtering and lift-off steps. Finally, we use SU-8 2002 (Kayaku Advanced Materials) to fabricate the insulating layer. The thickness of insulating layer is 2 *μ*m by spin coating at 3000 rpm following the product instruction. The thickness is confirmed by a surface profiler. A SU-8 insulating layer with 20 *μ*m opening ring is defined by photolithography to complete the Au-nanoroded device. The obvious boundaries can be found among the SU-8, ZnO nanorod, and Au nanorod regions (Figure 2(b)). The function of the SU-8 openings can form the sensing and regulating electrodes. A 2 *μ*m thick insulation layer of SU-8 is crossed with the metal-covered nanorod region to form 20 × 20 *μ*m^2^square nanoroded microelectrodes. As shown in Figure 2(c), the high-density ZnO nanorods with a length of ~700 nm and a diameter of ~100 nm vertically stand on the substrate. Following the conductive layer sputtering, the diameter of nanorods increases in contrast to that of the original ones (Figure 2(d) and Supplementary Figure S1). Figures 2(e)–2(g) show the layout of the Au-nanoroded microelectrode arrays. The device possesses the 32 recording electrodes and 2 reference electrodes (Figure 2(e)). The enlarged view illustrates the layout of the nanoroded region (purple) with a diameter of 4 mm and conductive recording electrode tracks with a width of 20 *μ*m at the center of device. The SU-8 openings are designed in the nanoroded recording electrode and reference electrode regions (Figure 2(f)). Intersections of the SU-8 opening ring and conductive tracks are 20 × 20 *μ*m^2^ recording sizes (Figures 2(g) and 2(h)).

Figure 2(i) shows the integrated Au-nanoroded biosensing and regulating system, including the He-Ne laser with a wavelength of 808 nm, Au-nanoroded device, and self-developed electrophysiological recording system. Due to the good absorption peak of the Au-nanoroded device in the near-infrared band at 800-900 nm, the 808 nm He-Ne laser is used to irradiate the device to produce the photothermal effect. The Au-nanoroded device is further assembled by cell culture or electrical components. The Au-nanoroded device unit is composed of a glass ring, Au-nanoroded electrode array chip, and printed circuit board (PCB) adapter from top to bottom (Figure 2(j) and Supplementary Figure S2). The glass ring with a diameter of 1.4 cm and a height of 1 cm is glued at the center of the Au-nanoroded electrode array chip as a cell culture chamber. The chip is then attached to the PCB adapter by PDMS, and all the electrode pads are electrically connected to PCB pads by a silver conductive glue. Pin headers are finally soldiered on the PCB adapter to match the interface of the self-developed electrophysiological recording system. Following the cardiomyocyte culture for days, the spontaneous extracellular potential of cardiomyocytes can be recorded by the Au-nanoroded device and self-developed electrophysiological system, and the He-Ne laser can generate a wavelength of 808 nm to irradiate on cells. Both electrophysiological signal recording and laser regulating can be dynamically performed during the whole experiment (Supplementary Figure S3). By laser irradiation, the photothermal effect is induced on the Au nanorods, and the temperature of the Au-nanoroded surface will increase (Figure 2(j)). By regulating laser irradiation with a power of 1.5 W/cm^2^ for 5 min, temperature of the Pt-nanoroded device is higher than that of the Pt-planar device. In the thermal images, the maximum temperature of Pt-nanoroded MEA is 39.2°C, which is 13.3°C higher than that of edge area. Meanwhile, the maximum temperature of Pt-planar MEA is 32.6°C, which is 9.9°C higher than that of the edge area (Supplementary Figure S4). Meanwhile, the maximum temperature of Au-nanoroded MEA is 41.3°C, which is 15.3°C higher than that of the edge area. Meanwhile, the maximum temperature of Au-planar MEA is 31.9°C, which is 9.5°C higher than that of the edge area (Figure 2(k)). Compared with the thermal images of different devices, the maximum temperature and temperature variation of Au-nanoroded MEA are higher and larger than the other three devices, demonstrating that the Au-nanoroded device possesses a good photothermal effect.

### 2.2. Bradyarrhythmia Modeling and Regulating on Au-Nanoroded Device

**Figure 3:**
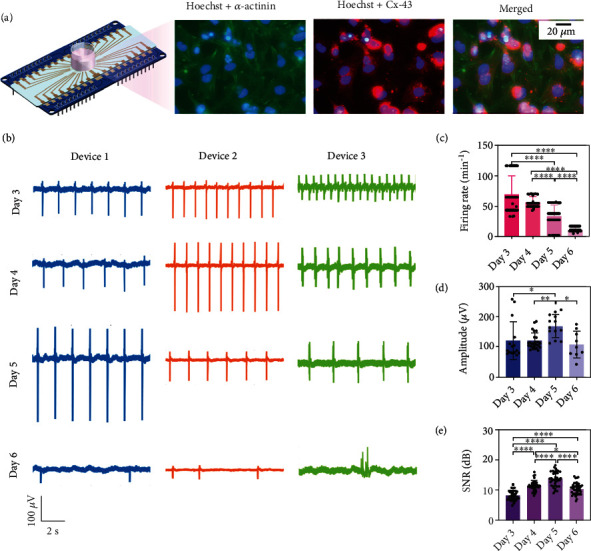
Bradyarrhythmia modeling and evaluating on the Au-nanoroded device. (a) Immunofluorescence images of cardiomyocytes cultured on the Au-nanoroded device. The *α*-actinin of cardiomyocytes is labeled by the primary antibody (mouse anti-*α*-actinin) and secondary antibody (Alexa Fluor 488 goat anti-mouse, green fluorescence). Cx-43 are stained by primary antibody (rabbit anti connexin-43) and secondary antibody (Alexa Fluor 568 donkey-anti-rabbit, red fluorescence), and the nucleus are stained by Hochest (blue fluorescence). (b) Extracellular electrical signal profiles of cardiomyocytes cultured on Au-nanoroded MEA with different cultured days (day 3 to day 6). (c) Statistical analysis of the firing rate, amplitude, and signal-to-noise ratio (SNR) of the bradyarrhythmia model. Significant differences are analyzed by ANOVA. *N* = 8; ^∗^*p* < 0.05, ^∗∗^*p* < 0.01, ^∗∗∗^*p* < 0.001, and ^∗∗∗∗^*p* < 0.0001.

The biocompatibility of Au-nanoroded device is first examined by immunostaining-cultured cardiomyocytes.  Figure 3(a) exhibits those cardiomyocytes grow and connect well with each other on the device, where Hochest (blue) labels the nuclei to recognize cells, and *α*-actinin (green) and Cx-43 (red) are labelled to characterize the cytoskeleton and connection, respectively. The immunostaining results demonstrate that the Au-nanoroded device possesses a good biocompatibility for cardiomyocyte growth compared to other nanoparticles such as TiO_2_ and CuO [45]. When cardiomyocytes are grown on the device and cultured for days, they are coupled with Au-nanoroded electrode, which can detect the electrical signal of cardiomyocytes which indicate the good biocompatibility of the device. It is common to reflect the biocompatibility or biosafety by electrophysiological signals in other studies [46–48]. The biocompatibility of cardiomyocytes can be demonstrated not only by fluorescent images but also is reflected by recording electrical signals. The cardiomyocytes can fire electrical signals in the preirradiation and postirradiation, indicating that the cardiomyocytes possess a good biocompatibility after the optimal photothermal regulation. To perform the photothermal regulation, the bradyarrhythmia is modelled on the Au-nanoroded device. It is well known that the signal firing rate of cardiomyocytes will increase to a high level at the beginning and gradually decrease to a low level following a long-term culture. To model the bradyarrhythmia, it is necessary to explore the suitable culture day for the following photothermal assays. The coupling of each chip to the cardiomyocytes will be slightly different due to the individual differences of cardiomyocytes, so the electrical signal profiles will be different.  Figure 3(b) presents the typical electrophysiological signals from three devices from day 3 to day 6. During day 3 to day 5, the cardiomyocytes generate the spontaneous and rhythmic extracellular potentials, while the firing rates are arrhythmic and dynamically decrease on day 6 in all the Au-nanoroded devices.

Cardiomyocytes are grown on the device for culture, and normal electrical signals can be detected by day 3 (Figure 3(c)). The signal firing rate of cardiomyocytes is between 68.4 min^−1^ at day 3 and 56.8 min^−1^ day 4, and then, the signal firing rate gradually slowed down to only 11.32 min^−1^ on day 6, which is ~80% lower than the normal firing rate, and the slower firing rate is considered the bradyarrhythmia model of cardiomyocytes. Meanwhile, the amplitude and SNR of extracellular potentials slightly change and present a high level on day 5 (168.6 *μ*V and 13.71 dB) as shown in Figures 3(d) and 3(e). The cardiomyocytes are gradually mature on the device. On day 5, the cardiomyocyte device coupling generally improves and sealing impedance increases, so that the amplitude of extracellular electrical signal is higher than before. Meanwhile, SNR refers to the ratio of the extracellular electrical signal to the device noise, so the SNR present high value when high extracellular electrical signal amplitude is detected based on the similar noise level. Among these parameters of cardiomyocytes, the firing rate is the key factor to evaluate the bradyarrhythmia model. Based on the analysis of electrophysiological signals, the cardiomyocytes present the bradyarrhythmia state on day 6, and the bradyarrhythmia model is successfully established after a 6-day culture and is ready to perform the following photothermal therapy.

### 2.3. Optimization of Photothermal Therapy on Bradyarrhythmia Model

**Figure 4:**
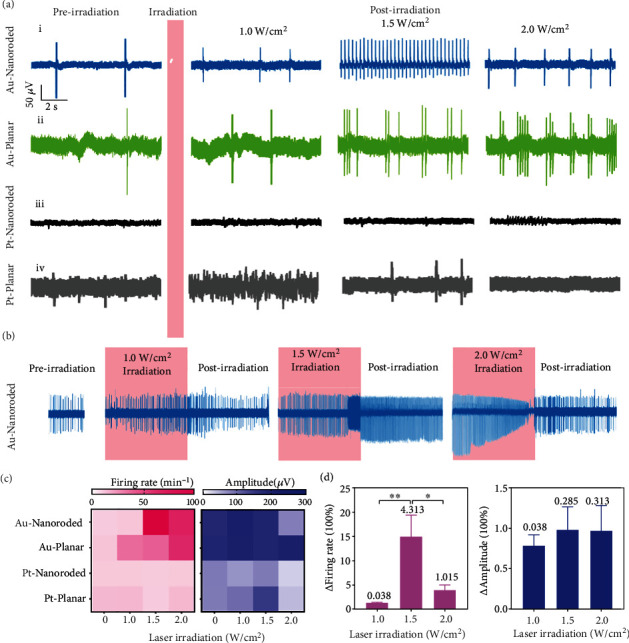
Photothermal effect on electrophysiological signals of bradyarrhythmia. (a) Typical electrophysiological signal profile of cardiomyocytes cultured on different devices (Au-nanoroded (A), Au-planar (B), Pt-nanoroded (C), and Pt-planar (D)) under different laser irradiations (1.0, 1.5, and 2.0 W/cm^2^ for 5 min). (b) Continuous electrophysiological signal recordings of cardiomyocyte during the whole photothermal regulating process. (c) Heat map of the firing rate and amplitude of cardiomyocytes under different conditions. (d) Statistical analysis of the firing rate and amplitude of cardiomyocytes cultured on Au-nanoroded device by the photothermal therapy with laser irradiation (1.0, 1.5, and 2.0 W/cm^2^ for 5 min). The average values of the standard deviations are labeled above the bar (*N* = 60). Significant differences are analyzed by ANOVA. ^∗^*p* < 0.05, ^∗∗^*p* < 0.01.

To investigate the photothermal treatment on cardiomyocytes with bradyarrhythmia, the NIR laser irradiation with a wavelength of 808 nm is regulated on the 6-day-cultured cardiomyocytes for 5 min, and irradiation is then stopped. Electrophysiological signals are continuously recorded in the whole process. Four types of devices (Au-nanoroded, Au-planar, Pt-nanoroded, and Pt-planar) and three types of irradiation power (1.0, 1.5, and 2.0 W/cm^2^) are all applied to optimize the conditions of photothermal regulation. The typical profiles of electrophysiological signals in the preirradiation, irradiation, and postirradiation stages are displayed as shown in Figures 4(a) and 4(b) and Supplementary Figure S5. The bradyarrhythmia models possess a low firing rate (below 16 min^−1^) after a 6-day culture in the preirradiation stage. In the cases of Au-nanoroded devices, laser irradiation with a power of 1.5 W/cm^2^ presented a better photothermal effect on the bradyarrhythmia model than other irradiation powers (Figure 4(a, A)). The signal firing rate of cardiomyocytes regulated by a power of 1.5 W/cm^2^ (98.0 min^−1^) is significantly higher than those of 1.0 W/cm^2^ (11.4 min^−1^) and 2 W/cm^2^ (68.3 min^−1^). As shown in Figure 4(a, B), the signal firing rate of cardiomyocytes gradually rises as the laser irradiation power increases (control: 11.5 min^−1^; 1.0 W/cm^2^: 35.0 min^−1^; 1.5 W/cm^2^: 38.8 min^−1^; and 2 W/cm^2^: 63.8 min^−1^); however, it remains the arrhythmic state. Meanwhile, the cardiomyocytes on Pt-nanoroded and Pt-planar devices have no obvious response to the photothermal regulation in the irradiation and postirradiation stages with the similar bradyarrhythmia state, respectively, implying a low absorbance of Pt-based devices at 808 nm NIR laser irradiation (Figure 4(a, C and D)). In addition, the noise of nanoroded device is lower than that of the planar device due to a large nanoroded surface area which induces the low electrode impedance.

Figure 4(b) and Supplementary Figure S5 displayed the continuous electrophysiological signal recordings of cardiomyocyte during the whole photothermal regulating process. The bradyarrhythmia model on Au-based devices can be effectively regulated by the 1.5 W/cm^2^ laser irradiation; the signal firing rate of cardiomyocytes increased at the irradiation stage. Following the removal of laser irradiation, the firing rate of the model on Au-nanoroded device continuously maintains at a high level (Figure 4(b)), while the signal firing rate of model on the Au-planar device returns to the bradyarrhythmia state as those in the preirradiation stage (Supplementary Figure S5 (ii)). Besides, the Pt-based devices present no photothermal regulating effect on cardiomyocytes under the 808 nm laser irradiation (Supplementary Figure S5 (iii) and (iv)). When the laser irradiation is at 2.0 W/cm^2^, cardiomyocytes respond rapidly during the irradiation interval. However, the cardiomyocytes receive a stronger photothermal regulating effect, which will also cause the damage to them. When the irradiation is stopped, the firing rate of the damaged cardiomyocytes dramatically decreased, so the 1.5 W/cm^2^ irradiation raised higher firing rate than 2.0. The treated cardiomyocytes with a high firing rate in the postirradiation stage are considered the effective photothermal regulation. To visually compare the photothermal repair effect with different conditions, the heat map is introduced to analyze the firing rate and amplitude of electrophysiological signals (Figure 4(c) and Supplementary Figure S6). In contrast to 0 W/cm^2^ as the control group (first column), the laser irradiation with a power of 1.5 W/cm^2^ shows a good photothermal repair effect based on the firing rate, and amplitudes of signals show no significant change under photothermal regulation. Based on our regulating conditions, we can conclude that the firing rate seldom change at the irradiation intensity of 1.0 W/cm^2^, while the firing rate is the highest at the irradiation intensity of 1.5 W/cm^2^, and then gradually decreases as the irradiation intensity increases. The amplitude of electrical signal has no significant change at irradiation intensity of 1.0-1.5 W/cm^2^, while the amplitude presents significant decrease at the irradiation intensity is 2.0 W/cm^2^. To evaluate the photothermal therapy, the change folds of the firing rate are derived and calculated under laser irradiation with different powers (Figure 4(d)). The signal firing rate of 1.5 W/cm^2^-treated cardiomyocytes is 15.1 times higher than that of original bradyarrhythmia and present a good rhythm. In contrast, the change folds of the firing rate under 1.0 and 2.0 W/cm^2^ treatment are only 1.4 and 4.0. The amplitude under different laser irradiations shows no obvious changes. Compared with the Au-planar, Pt-nanoroded, and Pt-planar substrate, the Au-nanoroded array has a high absorption at 808 nm based on its three-dimensional nanorod structure, which can accumulate enough heat and increase the temperature, so that the high temperature of the Au-nanoroded array can effectively stimulate to respond and make the cardiomyocytes with bradyarrhythmia return to the normal. All the results indicate that the photothermal effect of the Au-Nanoroded device under 1.5 W/cm^2^ 808 nm NIR laser irradiation is higher than that of other devices or laser powers, and the high firing rate can maintain for a long period of time in the postirradiation stage.

**Figure 5:**
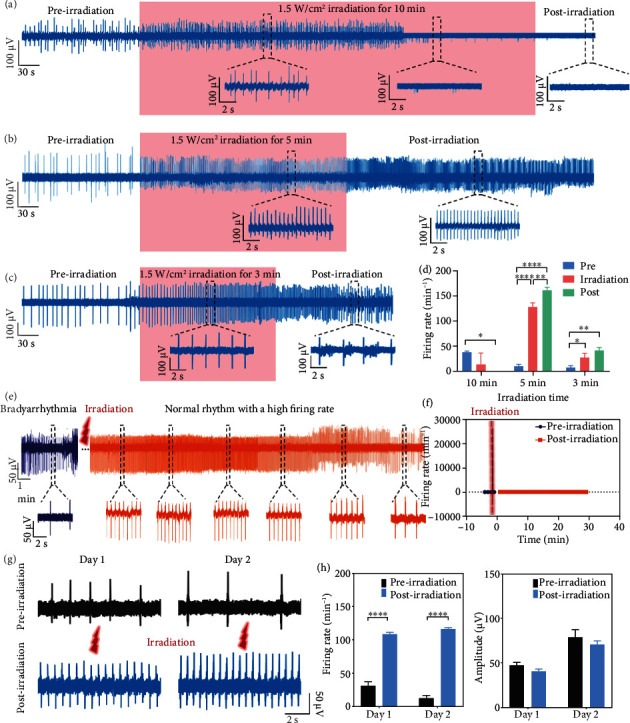
Optimization of photothermal regulating duration for the bradyarrhythmia model. (a–c) Typical electrophysiological evolution of cardiomyocytes with bradyarrhythmia by 10 min (a), 5 min (b), and 3 min (c) laser irradiation treatment. (d) Statistics of firing rate evolution of cardiomyocytes in the preirradiation, irradiation, and postirradiation stages. (e) Continuous extracellular electrical signal profile of cardiomyocytes cultured by Au-nanoroded MEA before and after the 1.5 W/cm^2^ laser irradiation. (f) Statistics of the firing rate for a long period of time. (g) Electrical signals of preirradiation and postirradiation from primary rat cardiomyocyte over consecutive two days. (h) Statistical histogram showing the firing rate and amplitude of signals changes from day 1 to day 2. Significant differences are analyzed by ANOVA. *N* = 8; ^∗^*p* < 0.05, ^∗∗^*p* < 0.01.

To improve the performance of photothermal regulating, duration of laser irradiation is further optimized to investigate the sustainability of photothermal therapy. Continuous recordings of bradyarrhythmia models begin at the preirradiation stage, and 1.5 W/cm^2^ laser irradiation is then applied on the model for 10 min. When the laser irradiation is regulated on the model, the firing rate of electrophysiological signals dramatically rises at the early irradiation stage; however, the signals are significantly inhibited due to the long laser irradiation at the late irradiation stage. The signals of model cannot recover at the postirradiation stage due to the irreversible cell damage (Figure 5(a)). To protect the cell viability, laser irradiation duration is reduced to 5 min for photothermal regulation. When irradiation is applied on the bradyarrhythmia model, the firing rate significantly increases during the whole 5 min irradiation and can continuously remain rhythmic for a long period of time in the post-irradiation stage (Figure 5(b)). To explore the performance of shorter irradiation duration, 3 min irradiation is applied on the bradyarrhythmia model, and the signal firing present similar evolution at the irradiation stage and can maintain a high firing rate for a short period of time at the early postirradiation stage, but rapidly fall into arrhythmia as shown in Figure 5(c). In our experiments, we use different devices simultaneously for parallel experiments. The coupling of cardiomyocyte electrode will be different, so the electrical signal profiles will be different. However, the bradyarrhythmia model of cardiomyocytes is based on the slower firing rate; therefore, the slight differences of the preirradiation firing rate among 10, 5, and 3 min irradiation duration will not affect the photothermal repair assay. From the statistical results, it can be concluded that the 5 min laser irradiation is optimized for Au-nanoroded device to perform the photothermal regulation and protect the cell viability (Figure 5(d)). Based on this optimized 5 min and 1.5 W/cm^2^ laser irradiation, the sustainability of the photothermal therapy is investigated. As shown in Figure 5(e), 1.5 W/cm^2^ laser irradiation is first administrated on the bradyarrhythmia model for 5 min and then stopped. The electrophysiological signals of the bradyarrhythmia model are continuously recorded in the whole process. Signal firing presents a sharp rise after irradiation and maintain the normal rhythm with a high firing rate over 20 min (Figure 5(f)). The amplitude of the signal seldom changes during the preirradiation and postirradiation periods (Supplementary Figure S7). Therefore, it is demonstrated that the optimized photothermal therapy can effectively treat the bradyarrhythmia in a noninvasive and label-free manner. Further, to investigate the sustainability of the photothermal regulation on cardiomyocyte repair, repeated irradiation is performed, and electrical signals are recorded. The laser irradiation of 1.5 W/cm^2^ for 5 min can well repair the cardiomyocyte without causing damage, thus achieving two consecutive days of electrical signal recording. As shown in Figure 5(g), cardiomyocytes can monitor the electrical signals after twice laser irradiation over two consecutive days. After irradiation, the firing rate of signals is increased from 31.66 ± 5.72 min^−1^ to 108.77 ± 2.51 min^−1^ on day 1 and 12.42 ± 4.45 min^−1^ to 116.54 ± 2.84 min^−1^ on day 2 (Figure 5(h)). While the amplitude of the signal seldom changed during the preirradiation and postirradiation periods in two consecutive days. Therefore, it demonstrated the multiple cycle irradiations when electrical signals can be detected to enhance the repair effect on the bradyarrhythmia.

### 2.4. Characterization of Photothermal Therapy by mRNA Sequencing

**Figure 6:**
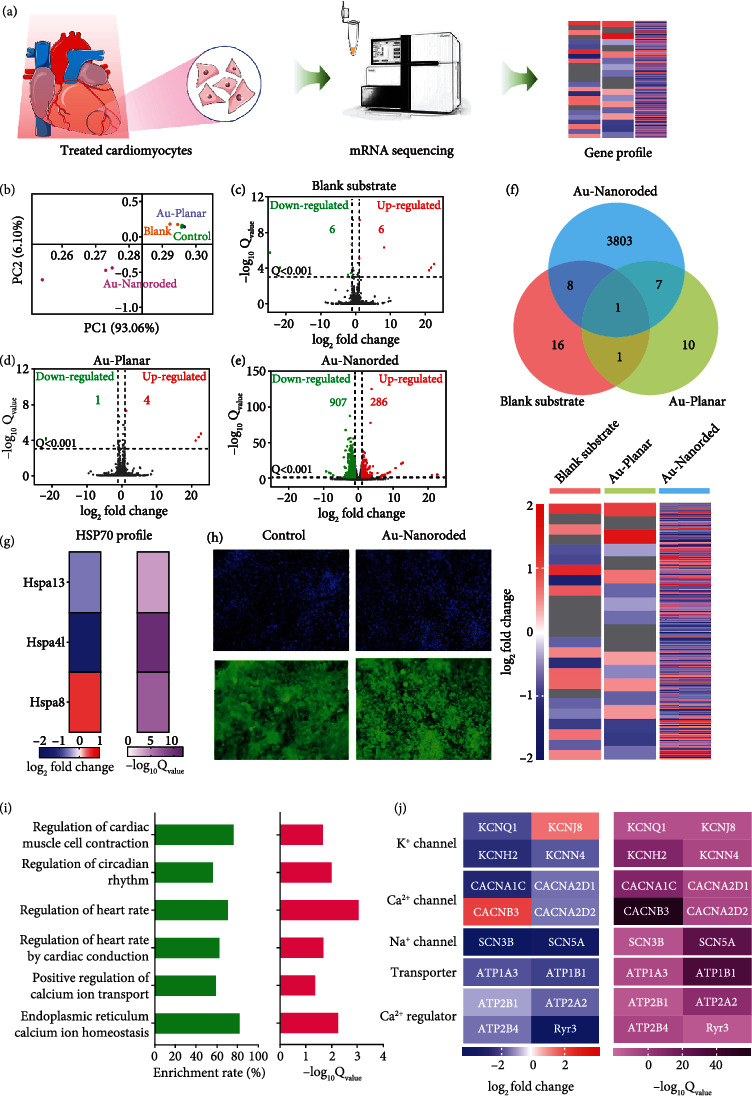
Photothermal regulation effect on cardiomyocytes characterized by mRNA sequencing. (a) Schematic diagram of mRNA sequencing. After treating with irradiation, cardiomyocytes are lysed and analyzed by mRNA sequencing. (b) Principal component (PC) plot indicates technical replicates in each goup are similar (*N* = 3). (c–e) Volcano plots indicates upregulated and downregulated genes whose *Q* value is <0.001 and ∣log2 |  fold change ≥ 1 in each group (blank substrate, Au-planar, and Au-nanoroded) . The number of genes that are upregulated (red) or downregulated (green) is shown in the each plot. (f) Venn plot shows the differential genes expression among different groups. Heat map displays the changes of differential gene expression (*Q* value < 0.001). (g) Heat map of HSP70 shows the related genes with different expression levels (*Q* value < 0.001). (h) Fluorescent images of nuclei and HSP70 in cardiomyocytes cultured on the blank substrate and Au-nanoroded substrate treated with laser irradiation. Significant changes of gene expression induced by photothermal therapy. (i) Enrichment rate and *Q*_value_ of genes in the enriched gene ontologies (GOs) which are highly related in regulation of cardiac muscle cell contraction, regulation of circadian rhythm, regulation of heart rate, regulation of heart rate by cardiac conduction, positive regulation of calcium ion transport, and endoplasmic reticulum calcium ion homeostasis. (j) Heat map of genes encoding ion channel including Na^2+^ channel, K^+^ channel, Ca^2+^ channel, transporter genes, and Ca^2+^ regulatory protein genes.

To further verify the photothermal regulation on cardiomyocytes with bradyarrhythmia, mRNA sequencing is introduced to analyze the gene expression of cardiomyocytes cultured on these different devices, including blank substrate, Au-planar substrate, and Au-nanorded substrate. Followng culture for 6 days, the cells on each device are treated with 808 nm NIR laser irradiation and then lysed to performe mRNA sequencing to analyze the differences in gene expression (Figure 6(a)). Sequencing shows that over 1.8 × 10^4^ genes generate the altered expressions in cardiomyocytes after exposed to the laser irradiation. The principal component (PC) map shows three technical replicates in each condition, allowing a reliable comparison of different treatments on the gene expression (Figure 6(b)). The laser irradiation-treated cardiomyocytes on the blank substrate and Au-planar substrate are close to the control group without the laser irradiation, indicating the similar gene expression. The treated cardiomyocytes on Au-Nanoroded substrate present the significantly different gene expression. As shown in Figures 5(c)–5(e), volcano plots for genes shows that are upregulated or downregulated depending on the each condition. Gene expressions are considered as significant difference with *Q*_value_ (corrected *P*_value_) < 0.001 and with ∣log_2_ fold change | ≥1. Compared with the control group without the laser irradiation, the differentially expressed genes (DEGs) number in cardiomyocytes cultured on the blank and Au-planar substrates under laser irradiation is only 12 and 5, respectively, indicating that the laser irradiation hardly affects the cardiomyocytes cultured on the blank substrate and Au-planar substrate (Figures 5(c) and 5(d)). However, cardiomyocytes cultured on the Au-nanoroded substrate present the dramatic changes of DEGs under laser irradiation treatment with 286 upregulated and 907 downregulated DEGs (Figure 5(e)). It provides evidence that the Au-nanoroded substrate can induce the photothermal effect by laser irradiation, which enables cardiomyocytes to recover a normal rhythm.

In addition, the Venn diagram shows gene sequencing after laser irradiation at 1.5 W/cm^2^ for 5 min. Compared with the control group, Au-nanoroded possesses ~3810 different gene changes, while the Au-planar device possesses ~10 different gene changes (Figure 6(f)), so the firing rates of cardiomyocytes on two devices are significantly different at optimized laser irradiation at 1.5 W/cm^2^ for 5 min, indicating the effective photothermal regulation on cardiomyocyte in the Au-nanoroded group. According to the analysis of the DEGs, the Au-nanoroded substrate can produce the effective photothermal regulation on cardiomyocytes in contrast to the Au-planar substrate. In addition, the heat shock protein 70 (HSP70) in cells can respond to external stimuli. As shown in Figures 6(g) and 6(h), genes involved in the regulation of heat shock protein 70 and fluorescent staining are significantly changed compared to the control group. As the main heat shock protein in the heat shock protein family, HSP70 is often highly expressed by the stimulated cells of biological organisms to protect the body and cells, so it can be used to verify the photothermal stimulation. In the former work, it was suggested that Au nanorod-assisted PPTT would show different efficacy responses with different expression levels of heat shock proteins [49]. In addition, it was verified that the activation of Au-NR at 808 nm near-infrared light can generate heat for the photothermal therapy and its introduction stimulated the regulation of cytoskeletal proteins [50]. Figure 6(g) shows the related genes encoding HSP70 protein have different expression levels by applying a filtering criterion of *Q*_value_ < 0.001. To intuitively observe the HSP70 response of cardiomyocytes to laser irradiation, HSP70 antibodies are immunostained to show the expression level (Figure 6(h)). In contrast to the protein expression of cardiomyocyte cultured on Au-nanoroded MEA without laser irradiation, the expression of heat shock protein significantly increases under laser irradiation, indicating the effective photothermal stimulation of cardiomyocytes on Au-nanoroded MEA.

To study the gene expression programs that regulates the rhythm recovery of cardiomyocytes after photothermal treatment, the expression of functional genes related to rhythm activities is further analyzed by gene ontology (GO). GO enrichment analysis show that DEGs greatly change in regulation of cardiac muscle cell contraction, regulation of circadian rhythm, regulation of heart rate, regulation of heart rate by cardiac conduction, positive regulation of calcium ion transport, and endoplasmic reticulum calcium ion homeostasis, whose enrichment rates all exceed 50% and *Q*_value_ is lower than 0.05 (Figure 6(i)). These enriched genes encoding ion channels and regulatory proteins including Na^2+^ channel, K^+^ channel, Ca^2+^ channel, transporter genes, and Ca^2+^ regulatory protein genes significantly enhance expression level (Figure 6(j)). The first class is ion channel genes including KCNQ1, KCNJ8, KCNH2 (encoding potassium voltage-gated channel); KCNN4 (encoding potassium calcium-activated channel); CaCNA1C, CaCNA2D1, CaCNB3, and CaCNA2D2 (encoding calcium voltage-gated channel); and SCN3B and SCN5A (encoding sodium voltage-gated channel) which present obvious changes in gene expression. Among them, KCNJ8 encodes the inwardly rectifying potassium channel subunit Kir6.1, which is an ATP-sensitive potassium channel and involves in cell electrical excitability. CaCNA2D1 and CaCNA2D2 are involved in regulating L-type calcium channel and calcium electrical activities in the heart, respectively. The second class is transporter genes, such as ATP1A3 and ATP1B1 (involved in encoding the Na^+^/K^+^ pump (Na^+^/K^+^-ATPase)), which maintain intracellular ion homeostasis. The third class is Ca^2+^ regulatory genes, such as ATP2B1, ATP2B4, ATP2A2, and Ryr3. ATP2B1 regulates intracellular calcium ion levels and shows the slight changes in gene expression. ATP2B4 is involved in the calcium transport of the ATPase plasma membrane calcium transporter, which plays a role in regulating calcium homeostasis and calcium-mediated signaling pathways. ATP2A2 encodes the sarcoplasmic reticulum Ca^2+^ ATPase, which is related with calcium transport in the heart. Based on the gene analysis, it is demonstrated that the photothermal therapy are effectively regulated on bradyarrhythmia.

## 3. Conclusions and Perspectives

In this work, we develop an integrated Au-nanoroded biosensing and regulating platform to investigate the photothermal therapy of cardiac bradyarrhythmia *in vitro*. It is demonstated that the Au-nanoroded electrode array possess both signal recording and regulating functions. Bradyarrhythmia *in vitro* can be modelled after culture for a long time. After checking the signal firing rate of cardiomyocytes under different levels of laser irradiation, 1.5 W/cm^2^ laser irradiation for 5 min is optimized for cardiomyocytes cultured on Au-nanoroded MEA, and the firing rate can return to normal after irradiation and maintain for a long period of time. In contrast to optogenetic methods used to stimulate cardiomyocytes in previous studies, this nongenetic regualting method proposed in this work exclude the complex transfection steps. Photothermal stimulation of cardiomyocytes supported by Au-nanoroded takes advantage of the local thermal effects of the laser and Au-nanoroded interaction to accumulate enough thermal energy to restore rhythm of cardiomyocytes, providing a convenient method to treat bradyarrhythmia. Furthermore, mRNA sequencing shows that the DEGs in cardiomyocytes are significantly increased after photothermal regulation, mainly focusing on ion channel genes, transporter genes, and Ca^2+^ regulatory protein genes, which are related to the regulation of heart rate, signal transduction, and ion transport. This work provides the reliable evidence of photothermal repair on cardiomyocytes with bradycarrhythmia and proposes a high-efficiency biosensing and regulating platform to treat the bradycarrhythmia *in vitro*, which presents a high potential for cardiological clinical studies.

## 4. Materials and Methods

### 4.1. Device Fabrication and Assembly

For the device fabrication, 75 mm × 75 mm, 1 mm thick microscope slide (CITOTEST, China) is used as the substrate. A 4 mm diameter circle opening is fabricated by photolithography which is spin-coated by 2.5 *μ*m thick RZJ-390PG-50 photoresist (Ruihong Electronic Chemical, China), soft baked at 100°C for 2 min, exposed by I-line masker alinger (ABM, USA) at the dose of 300 mJ/cm^2^, and developed for 35 s using RZX3038 developer (Ruihong Electronic Chemical, China). The substrate is then sputtered with 1 nm thick ZnO in this 4 mm diameter circle opening using VTC 300 magnetron sputter as the seeds of ZnO nanorods (Microtech, China). ZnO nanorods are synthesized by hydrothermal method with 35 mM Zn(NO_3_)_2_·6H_2_O (Sigma, USA) and 35 mM Hmethyltetramine (C_6_H_12_N_4_) (Sigma, USA) aqueous solutions at 90°C for 2.5 h. The substrate is rinsed by deionized water after the synthesis of ZnO nanorods. Subsequently, the conductive layer is fabricated by the same photolithography protocol of RZJ-390PG-50 photoresist, Cr/Au or Cr/Pt (10 nm/100 nm) electrode, lead and pad are patterned by sputtering and lift-off steps. Part of the nanoroded region is covered by the Cr/Au or Cr/Pt pattern. Finally, a 2 *μ*m think insulation layer (Kayaku Advanced Materials, USA) is patterned by SU-8 2002, which is spin-coating at 3000 rpm/min, soft baking at 95°C for 1 min, exposed by an I-line masker aligner at the dose of 120 mJ/cm^2^, post baked at 95°C for 1 min, developed in propylene glycol methyl ether acetate (PGMEA) for 1 min, rinsed with isopropyl alcohol for 1 min, dried with N_2_ air gun, and hard baked at 150°C for 1 h on a hot plate to finish the device fabrication.

The device unit is assembled by a glass ring, Au-nanoroded electrode array chip, and printed circuit board (PCB) adapter, from top to bottom. The glass ring with a diameter of 1.4 cm and a height of 1 cm is glued at the center of MEA as a cell culture chamber by polydimethylsiloxane (PDMS) (Sylgard 184, Dow Corning, USA). MEA is attached to the PCB adapter by PDMS, and all the electrode pads are electrically connected to PCB pads by silver conductive glue (Electrolube, UK). Pin headers are finally soldiered on the PCB adapter to match the interface of self-developed electrophysiological recording system.

### 4.2. Scanning Electron Microscope (SEM) Characterization

Prior to SEM imaging, the device is fixed on the sample stage with conductive carbon tape and then sputtered with 2 nm gold to improve conductivity by VTC 300 sputter (Microtech, China). Scanning electron microscopy images are taken by SUPRA 60 SEM system (Zeiss, Germany) at an angle of 45°.

### 4.3. Primary Cardiomyocyte Culture

Devices are sterilized by 75% ethanol under UV radiation for 2 hours in the biosafety cabinet, and the devices are then coated with 10 *μ*g/ml fibrin solution (Sigma, USA) in a 4°C refrigerator overnight to improve cell adhesion. The primary cardiomyocytes are derived from 1 to 3 days old neonatal Sprague-Dawley rats (SPF, Laboratory Animal Center of Sun Yat-sen University). After the rats are sterilized by 75% alcohol, ventricular tissue is rapidly excised and immersed in the ice-cold Dulbecco's Modified Eagle Medium (DMEM, Thermo Fisher Scientific, USA). The tissues are cut into ~1 mm^3^ fragments in the cold Hanks' Balanced Salt Solution (HBSS, Thermo Fisher Scientific, USA). The fragments are then digested in HBSS dissolved with 0.07% trypsin (Thermo Fisher Scientific, USA) and 0.05% collagenase Type II (Thermo Fisher Scientific, USA). Each digestion step takes 8 min for 12 times in 37°C 5% CO_2_ incubator. After digestion, the cell suspension is collected in DMEM supplemented with 10% fetal bovine serum (FBS, Invitrogen, Carlsbad, CA) and centrifuged with 1000 rpm for 5 min. The resuspended cell suspension is filtered by 70 *μ*m cell strainer (Sigma, USA). By 45 min differential adhesion twice, cardiomyocytes are cultured in the device at a total number of 2.5 × 10^5^ cells/cm^2^ and cultured in 37°C and 5% CO_2_ incubator. The medium is replaced every two days to maintain sufficient nutrients. All protocols complied with regulations of Sun Yat-sen University Institutional Animal Care and Use Committee (approval no. SYSU-IACUC-2020-B0007).

### 4.4. Immunofluorescence Staining

After culture for 3 days, cardiomyocytes are performed immunofluorescence staining to check the state. The cells are washed with 0.01 M PBS and fixed with 4% paraformaldehyde for 30 min at room temperature. After 3 ice-cold PBS washes, samples are permeated with PBS containing with 0.25%Triton X-100 for 10 min at room temperature. Following 3 buffer washes, samples are incubated with a blocking solution of 1% BSA in PBST (PBS+0.1% Tween 20) for 30 min. After that, samples are incubated by diluted primary antibodies (mouse anti-*α*-actinin, 1 : 500, sc-17829, Santa, USA, and rabbit anti connexin-43, 1 : 200, ab11370, Abcam, UK) solution for 1 h at 37°C incubator. After incubation of primary antibodies, the samples are washed and incubated with 1 : 500 dilution of secondary antibodies in blocking solution: Alexa Fluor 488 goat anti-mouse (ab150117, Abcam, UK) and Alexa Fluor 568 donkey-anti-rabbit (ab175693, Abcam, UK) for another 1 hour. Following 3 buffer washes, nucleus is stained by Hoechst 33342 (Sigma, USA) for 5 min. All fluorescence images are performed with a fluorescence microscope (Leica, Germany).

### 4.5. Photothermal Regulating

Photothermal regulation is performed after cardiomyocytes are cultured for six days with bradyarrhythmia. The He-Ne laser with a wavelength of 808 nm is first turned on to preheat for 5 min, and the parameters of laser power (1.0, 1.5, and 2.0 W/cm^2^) are set for irradiation. The MEA device with cultured cardiomyocytes is aligned then under the laser and irradiated for a given time, while the extracellular potential of cardiomyocytes is simultaneously recorded in real time.

### 4.6. Electrophysiological Signal Recording

After the cardiomyocytes exhibited spontaneous beating (usually 3 days after culture), the nanoroded electroeds is connected to a self-developed 32-channel electrophysiological measurement system for extracellular potential recordings at 20 kHz sampling rate. The signals are then filtered with a bandpass of 1 Hz–7.6 kHz by hardware. All the experiments can be performed in 37°C incubator.

### 4.7. mRNA Sequencing

The cardiomyocytes are washed with PBS and lysed by TRIzol solution (ThermoFisher, USA). The lysate is transferred into centrifugal tube of RNase-free (ThermoFisher, USA), stored at -80°C, and sent company (BGI Group) for mRNA sequencing. Data analysis is based on interactive software Dr. Tom by BGI Group. Differential genetic detection adopts DEGSeq method, which is proposed a new method based on MA-Plot, which is a widely used statistical analysis tool for chip data. To improve the accuracy of DEGSeq, genes with ∣log_2_ |  fold change ≥ 1 and *Q* value <0.001 are defined and screened as significantly differentially expressed genes (DEGs). According to the results, the union of DEGs is performed by using R-package heat map for hierarchical clustering analysis. Functional classification of DEGs is conducted according to Gene Ontology annotation results and official classification. Meanwhile, Phyper function in R software is used for enrichment analysis and the *Q* value is calculated.

### 4.8. Statistical Analysis

The signal processing is performed by self-developed MATLAB 2020 program. All results and error bars are expressed as mean ± SD (standard deviation). The statistical analysis was performed using Prism 8.0 (GraphPad, USA) or Excel 2019 (Microsoft, USA).

## Data Availability

All data needed in the paper are present in the paper and in the supplementary section. Additional data related to this paper may be requested from the authors.

## Supplementary Materials

Supplementary Materials The authors provide supplementary information that offers details on some of the approach in this paper.
