# Supplementary material for: Integrated Au-Nanoroded Biosensing and Regulating Platform for Photothermal Therapy of Bradyarrhythmia
Source: Research (Wash D C). 2022 Feb 7;2022:9854342. doi: 10.34133/2022/9854342 (PMC8848336; doi:10.34133/2022/9854342)
Supplement: Supplementary Materials — The authors provide supplementary information that offers details on some of the approach in this paper. [file 9854342.f1.docx]

**Supplementary Information**

**Integrated Au-Nanoroded Biosensing and Regulating Platform for Photothermal Therapy of Bradyarrhythmia**

**Jiaru Fan****g^1,2,^**^+^**, Dong Liu^1,+^, Dongxin Xu^1,+^,** **Qianni Wu^1,^**^+^**, Hongbo Li^1^, Ying Li^3^, and Ning Hu^1,2,4*^**

^1^ State Key Laboratory of Optoelectronic Materials and Technologies, Guangdong Province Key Laboratory of Display Material and Technology, School of Electronics and Information Technology, State Key Laboratory of Ophthalmology, Zhongshan Ophthalmic Center, Guangdong Provincial Key Laboratory of Ophthalmology and Visual Science, Sun Yat-sen University, Guangzhou 510006, China.

^2^ ZJU-Hangzhou Global Scientific and Technological Innovation Center, Hangzhou, 311215, China.

^3^ Molecular Cancer Research Center, School of Medicine, Sun Yat-sen University, Shenzhen 518107, China

^4^ State Key Laboratory of Transducer Technology, Chinese Academy of Sciences, Shanghai 200050, China

^+^These authors contribute equally to this work.

^*^To whom correspondence may be addressed. Corresponding to: Ning Hu, [huning3@mail.sysu.edu.cn](mailto:huning3@mail.sysu.edu.cn).


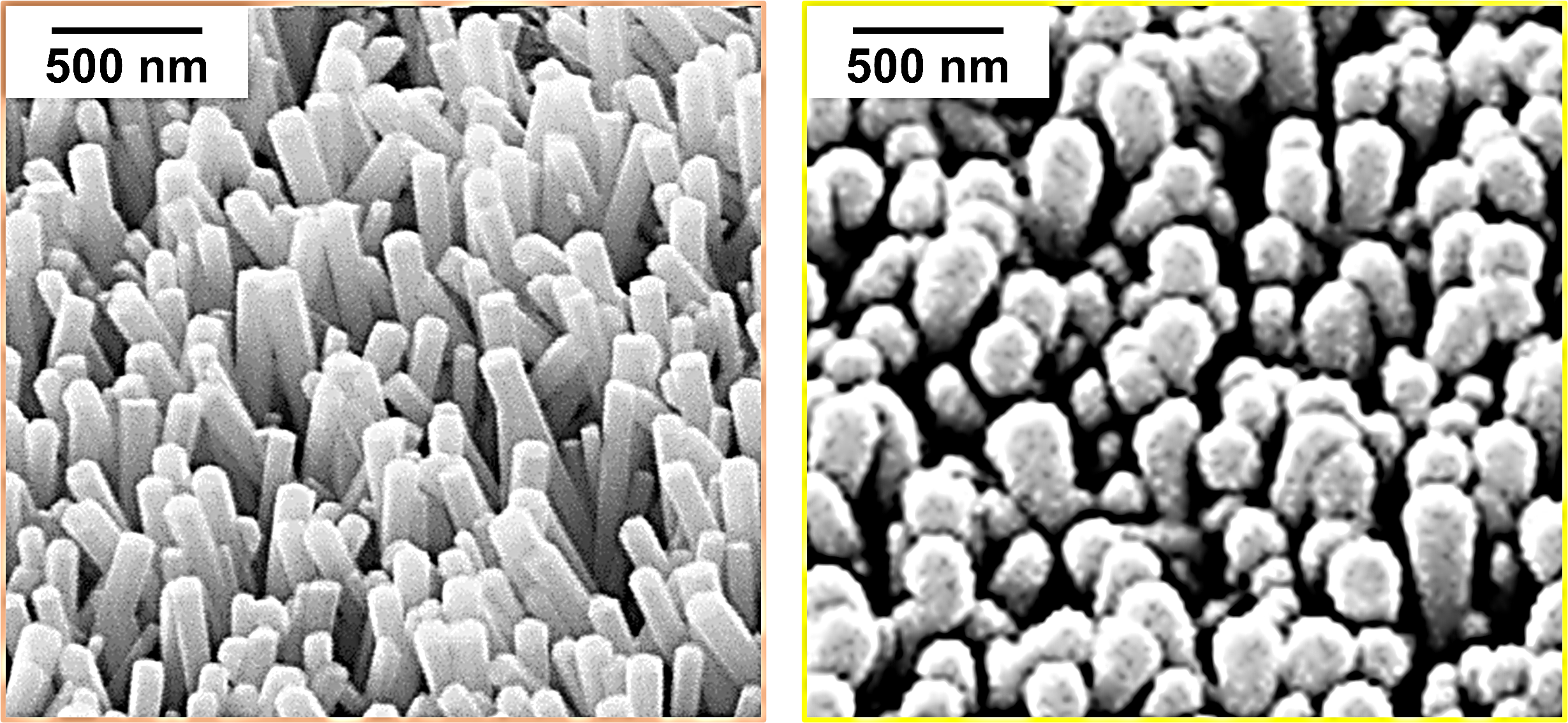


**Figure S1.** High-magnification SEM images of ZnO nanorods (Left panel) and the Au nanorods (Right panel).

**
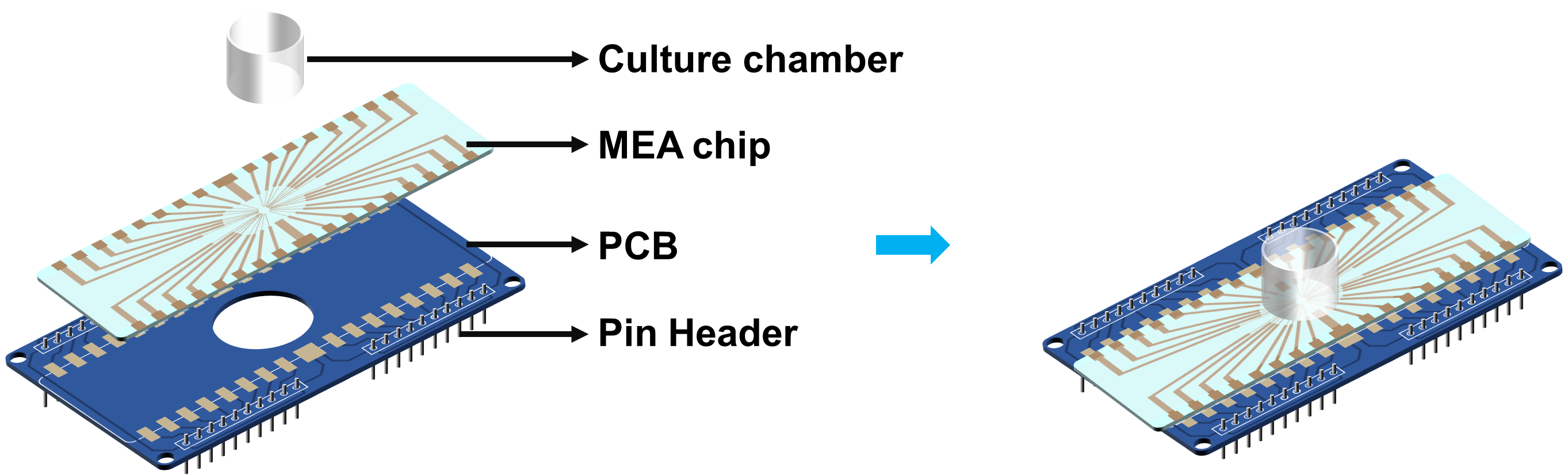
**

**Figure S2.** Device assembly diagram with cell culture chamber, nanoroded microelectrode array, printed circuit board (PCB), and pin header.


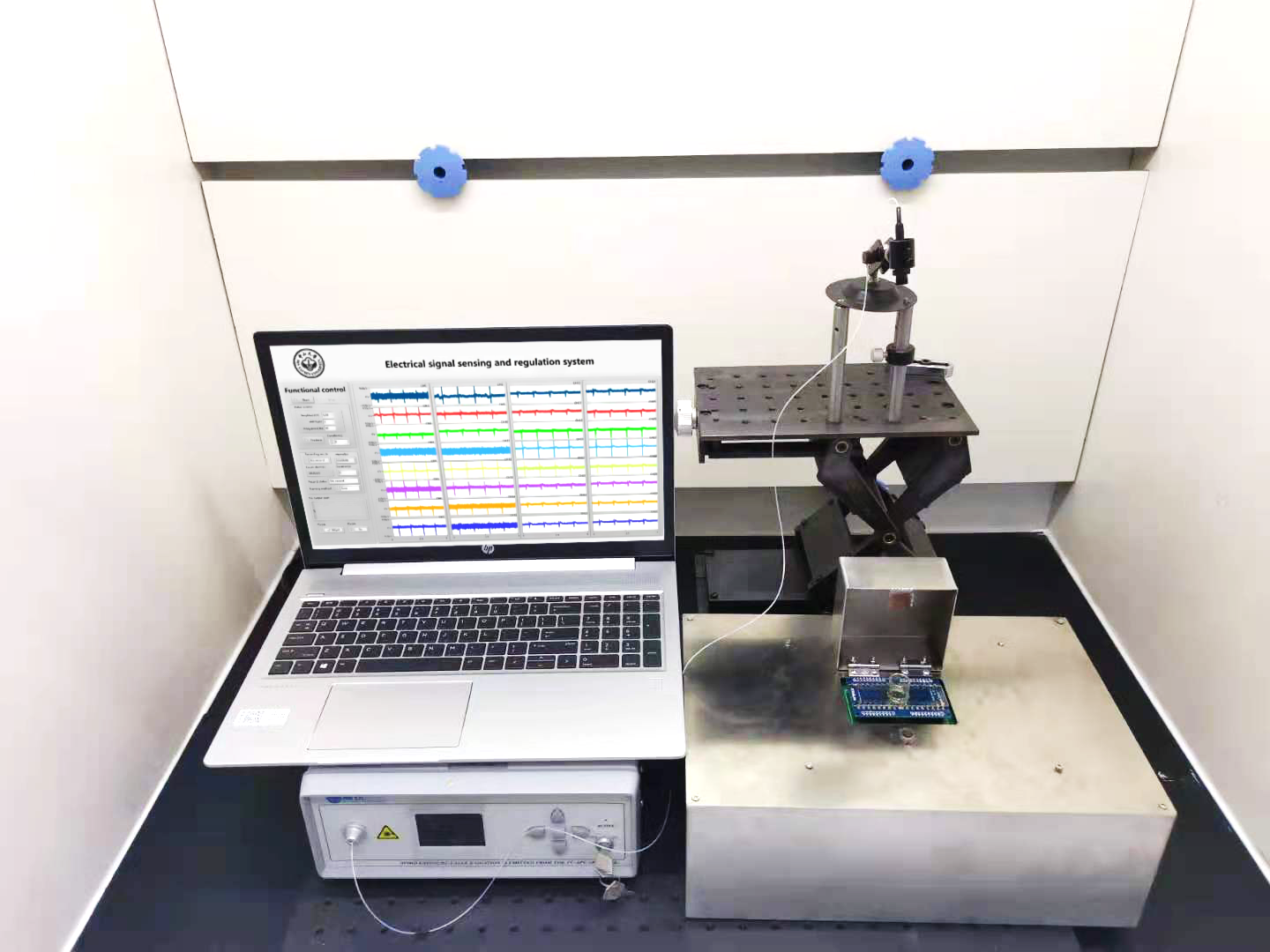


**Figure S3. Integrated Au-Nanoroded biosensing and regulating system.** The system consist of 808 nm fiber coupled laser, Au-Nanoroded MEA device, and self-developed electrophysiological recording system


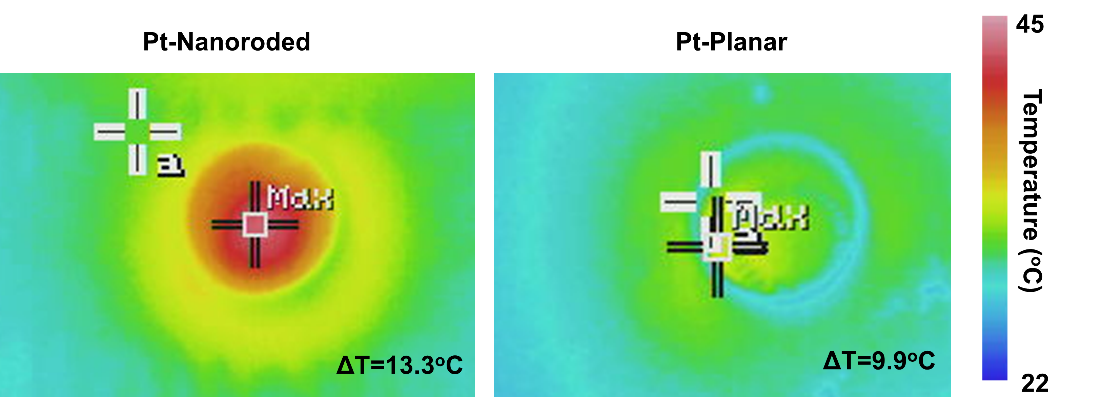


**Figure S4.** Thermal images of Pt-Nanoroded electrode array (MEA) and Pt-Planar MEA showing the temperature change after radiation with a power of 1.5 W/cm^2^ for 5 min. The maximum temperature of Pt-Nanoroded MEA is 39.2^o^C, which is 13.3^o^C higher than that of edge area. The maximum temperature of Pt-Planar device is 32.6^o^C, which is 9.9^o^C higher than that of edge area.


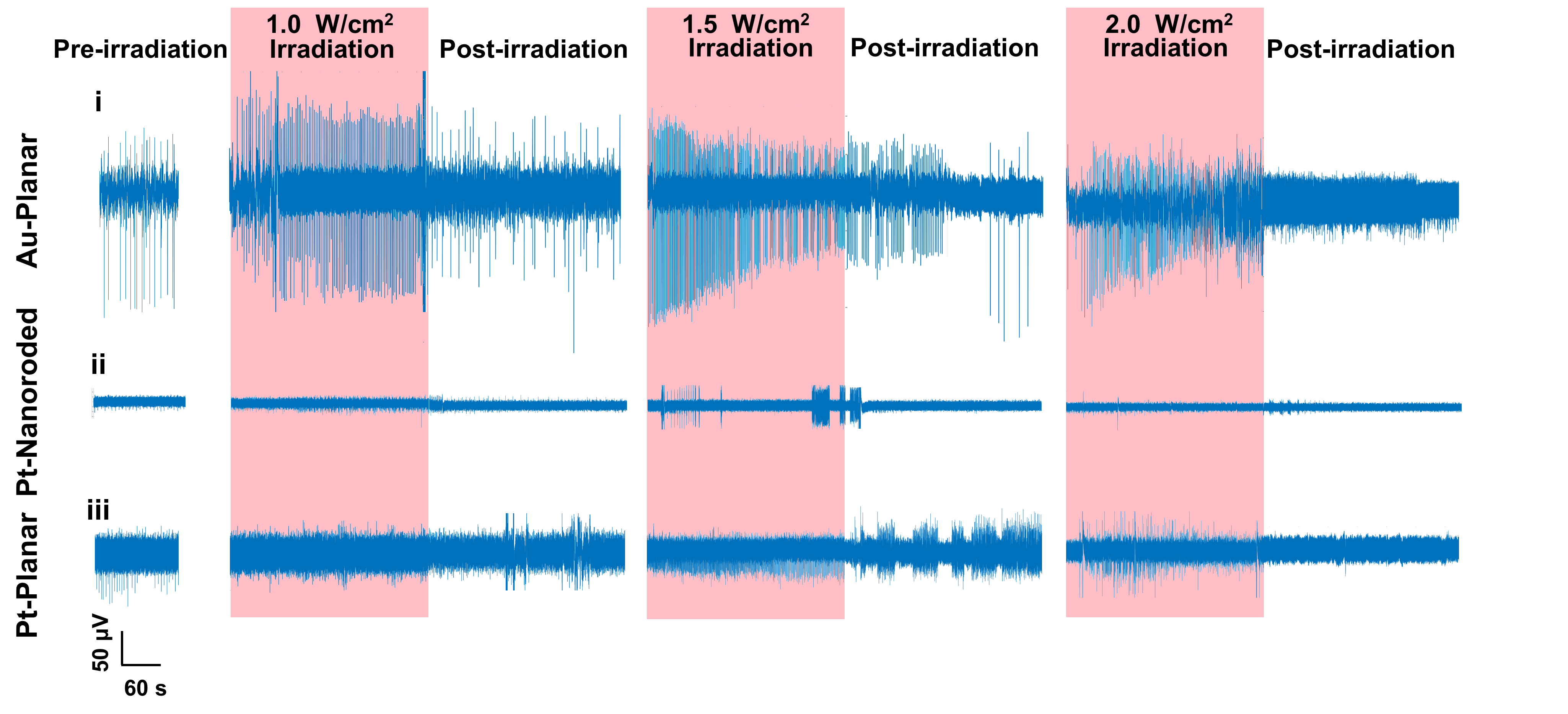


**Figure S5.** Typical electrophysiological profiles of cardiomyocytes cultured on different types of devices (Au-Planar (i), Pt-Nanoroded (ii), Pt-Planar (iii)) under NIR irradiation with different power density (1.0, 1.5, and 2.0 W/cm^2^ for 5 min) at the pre-irradiation, irradiation, and post-irradiation stages.

**Figure S6. Line chart of correlation between firing rate, amplitude and irradiation intensity.** The pink line is the correlation between firing rate and irradiation intensity, while the blue line is the correlation between amplitude and irradiation intensity


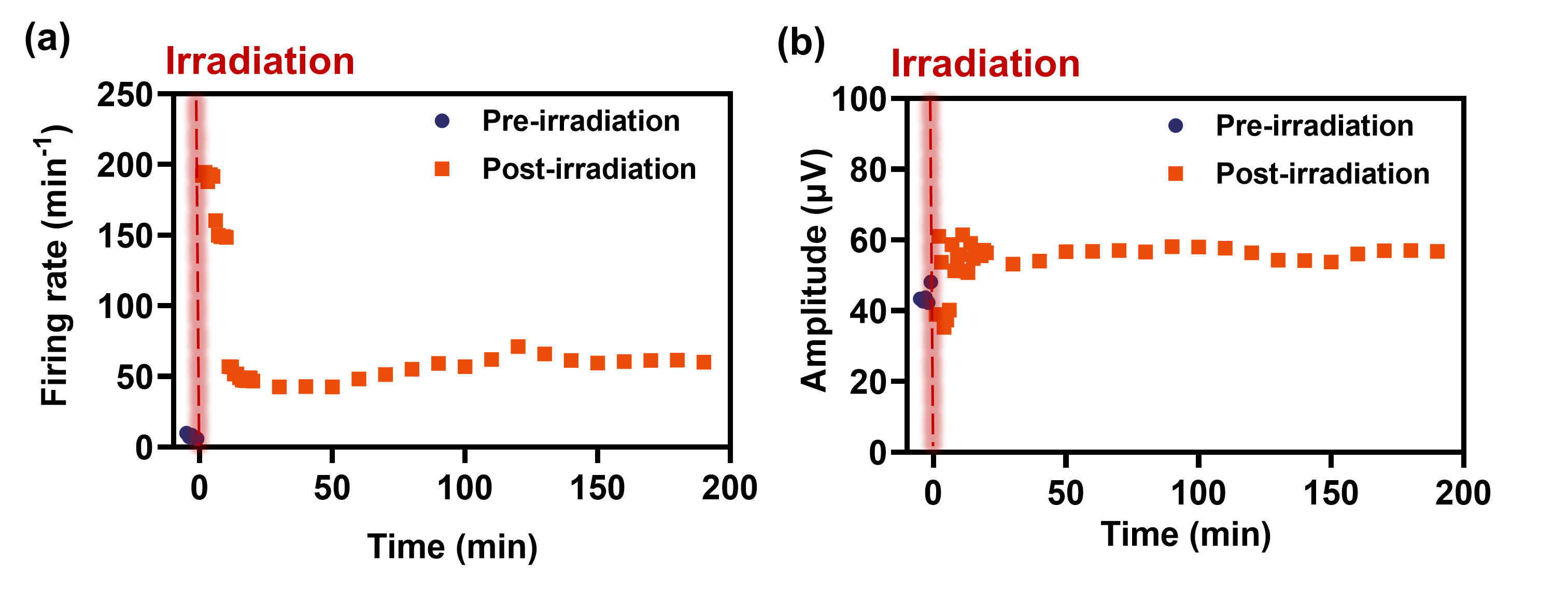


**Figure S7**. **Change in electrophysiological signal during continuous recordings by NIR irradiation with a power density of 1.5 W/cm^2^ for 5 min.** (a) Evolution of Firing rate, and (b) Amplitude.
